# Effectiveness of Pre-exposure Prophylaxis (PrEP) in the Prevention of Human Immunodeficiency Virus (HIV): A Systematic Review of Randomized Controlled Trials With Narrative Synthesis

**DOI:** 10.7759/cureus.105881

**Published:** 2026-03-26

**Authors:** Bhavesh Sharma, Ojasvi Sharma

**Affiliations:** 1 Medical School, University of Manchester, Manchester, GBR; 2 Ophthalmology, University Hospital Hairmyres, East Kilbride, GBR

**Keywords:** adherence, cabotegravir, global health, hiv prevention, hiv transmission, pre-exposure prophylaxis (prep), randomized controlled trials

## Abstract

Human immunodeficiency virus (HIV) prevention has advanced substantially, yet ending new transmissions by 2030 remains uncertain. Pre-exposure prophylaxis (PrEP) has high biological efficacy, but real-world effectiveness varies due to adherence, access, and delivery barriers.

This review aims to synthesize evidence on the efficacy of PrEP in reducing HIV incidence and to discuss its implications for achieving the Joint United Nations Programme on HIV/AIDS (UNAIDS) 2030 targets.

Following the Preferred Reporting Items for Systematic Reviews and Meta-Analyses (PRISMA) 2020 guidance, MEDLINE, Embase, Scopus, and Cochrane Library were searched for English-language studies published between January 2010 and January 2025 assessing PrEP efficacy in high-risk populations.

Nine randomized controlled trials (RCTs) comprising more than 20,000 participants across multiple continents were included. Across oral tenofovir-based PrEP trials, risk reduction ranged from 49% to 86% in intention-to-treat (ITT) analyses, with markedly higher efficacy in adherence-defined subgroups (e.g., up to 92-99% in trials with pharmacokinetic confirmation). Long-acting injectable cabotegravir (CAB-LA) was superior to daily oral tenofovir disoproxil fumarate-emtricitabine (TDF-FTC) in two large RCTs, with relative risk reduction up to 89% and high observed adherence consistent with clinic-based dosing. Adverse events were generally mild to moderate (gastrointestinal symptoms, headache), with expected renal marker changes in some oral PrEP trials and injection-site reactions for CAB-LA; no consistent evidence of risk compensation was observed.

PrEP is highly effective when taken as prescribed, but achieving population-level impact aligned with UNAIDS targets requires scale-up, improved access, and interventions that support persistence and adherence, including expanded delivery models and longer-acting options.

## Introduction and background

Human immunodeficiency virus (HIV) remains a major global public health challenge. In 2023, an estimated 1.3 million people acquired HIV, and approximately 39.9 million were living with HIV [1]. Sub-Saharan Africa continues to account for a substantial proportion of global incidence [2]. Although antiretroviral therapy (ART) coverage has expanded, ongoing infections indicate that prevention efforts remain insufficient [3]. The prevention toolkit includes ART for treatment and prevention (including the "Undetectable = Untransmittable" principle), condoms, testing, and biomedical prophylaxis [4].

Pre-exposure prophylaxis (PrEP) is a biomedical prevention strategy for HIV-negative individuals at increased risk of acquisition [5]. Oral tenofovir-based PrEP (tenofovir disoproxil fumarate (TDF) with or without emtricitabine (FTC)) was first approved in 2012 and demonstrated efficacy in multiple randomized trials among men who have sex with men (MSM), people who inject drugs (PWID), and heterosexual populations, including serodiscordant couples [5-7]. More recently, long-acting injectable cabotegravir (CAB-LA) has expanded the range of PrEP options and may reduce adherence barriers inherent to daily pill-taking [8,9].

The Joint United Nations Programme on HIV/AIDS (UNAIDS) has set an ambition to end acquired immunodeficiency syndrome (AIDS) as a public health threat by 2030 [10]. Understanding how PrEP performs across populations and settings, particularly the gap between biological efficacy and real-world effectiveness, is critical for informing policy, implementation strategies, and investment decisions. In this context, efficacy refers to the protective effect of PrEP under controlled clinical trial conditions, whereas effectiveness reflects the degree of protection achieved in real-world implementation settings where adherence, access, and health-system factors influence outcomes.

This review aims to summarize evidence on PrEP efficacy for preventing HIV acquisition across key populations, describe how adherence influences observed effectiveness, compare efficacy across PrEP modalities, including CAB-LA and oral regimens, and discuss implications for achieving UNAIDS 2030 prevention targets. While previous reviews have demonstrated the efficacy of PrEP across various populations, many have focused on specific populations or modalities and have not explicitly examined the gap between biological efficacy observed in controlled trials and real-world effectiveness influenced by adherence, access, and implementation barriers. This review addresses this gap by synthesizing randomized controlled trial (RCT) evidence alongside adherence-related outcomes to better understand how efficacy translates into population-level impact relevant to UNAIDS 2030 targets.

## Review

Materials and methods

Search Strategy

A structured search was performed in MEDLINE, Embase, Scopus, and Cochrane Library for studies published between January 2010 and January 2025 in English. Titles and abstracts were screened by the primary reviewer, followed by full-text eligibility assessment. Study selection decisions and eligibility criteria were discussed with a senior academic supervisor to ensure consistency with the predefined inclusion criteria and to minimize potential selection bias. Duplicates were removed using a reference management software (Zotero, Corporation for Digital Scholarship, Vienna, Virginia, United States) [11].

The search strategy combined controlled vocabulary terms and keywords related to HIV prevention and PrEP. Search terms included "HIV", "pre-exposure prophylaxis", "PrEP", "tenofovir", "emtricitabine", and "cabotegravir". Medical Subject Headings (MeSH) and database-specific indexing terms were used where applicable. Boolean operators were applied to combine concepts (e.g., "HIV AND pre-exposure prophylaxis", "PrEP OR tenofovir prophylaxis"). The final search was conducted in January 2025. Reference lists of relevant reviews and trials were also screened to identify additional eligible studies.

This systematic review was conducted and reported in accordance with the Preferred Reporting Items for Systematic Reviews and Meta-Analyses (PRISMA) 2020 guidelines [12].

Eligibility Criteria

Eligibility criteria were defined using the Population/Intervention/Comparison/Outcomes/Study design (PICOS) framework, a structured approach widely used to formulate systematic review inclusion criteria [12]. Populations included MSM, PWID, female sex workers/women in high-risk regions, and homosexual or heterosexual couples (including serodiscordant couples). Interventions included daily oral tenofovir-based PrEP regimens (TDF/FTC or tenofovir alafenamide (TAF)/FTC), event-driven PrEP (2-1-1), and CAB-LA. Comparators included placebo, no PrEP/deferred PrEP, alternative PrEP regimens, or standard prevention. Outcomes of interest included HIV incidence reduction, adherence, risk compensation, and adverse effects. Eligible designs included RCTs and selected comparative regimen trials.

Studies were excluded if they involved non-human populations, general populations without HIV risk stratification, or HIV-positive individuals receiving treatment-focused ART. Interventions limited to post-exposure prophylaxis (PEP), ART-only strategies, or mixed prevention studies without clear tenofovir-based PrEP-specific outcome reporting were excluded. Studies without a comparator group, those comparing ART or sexually transmitted infection (STI) treatment regimens unrelated to PrEP, or those failing to report relevant clinical outcomes were omitted. Additionally, narrative-only reviews without primary or secondary data, studies assessing behavioral intention or awareness without actual PrEP usage data, case reports, non-systematic reviews, editorials, commentaries, opinion pieces, conference abstracts without full data, non-English publications, studies published before 2010, and articles without accessible full text were excluded.

**Figure 1:**
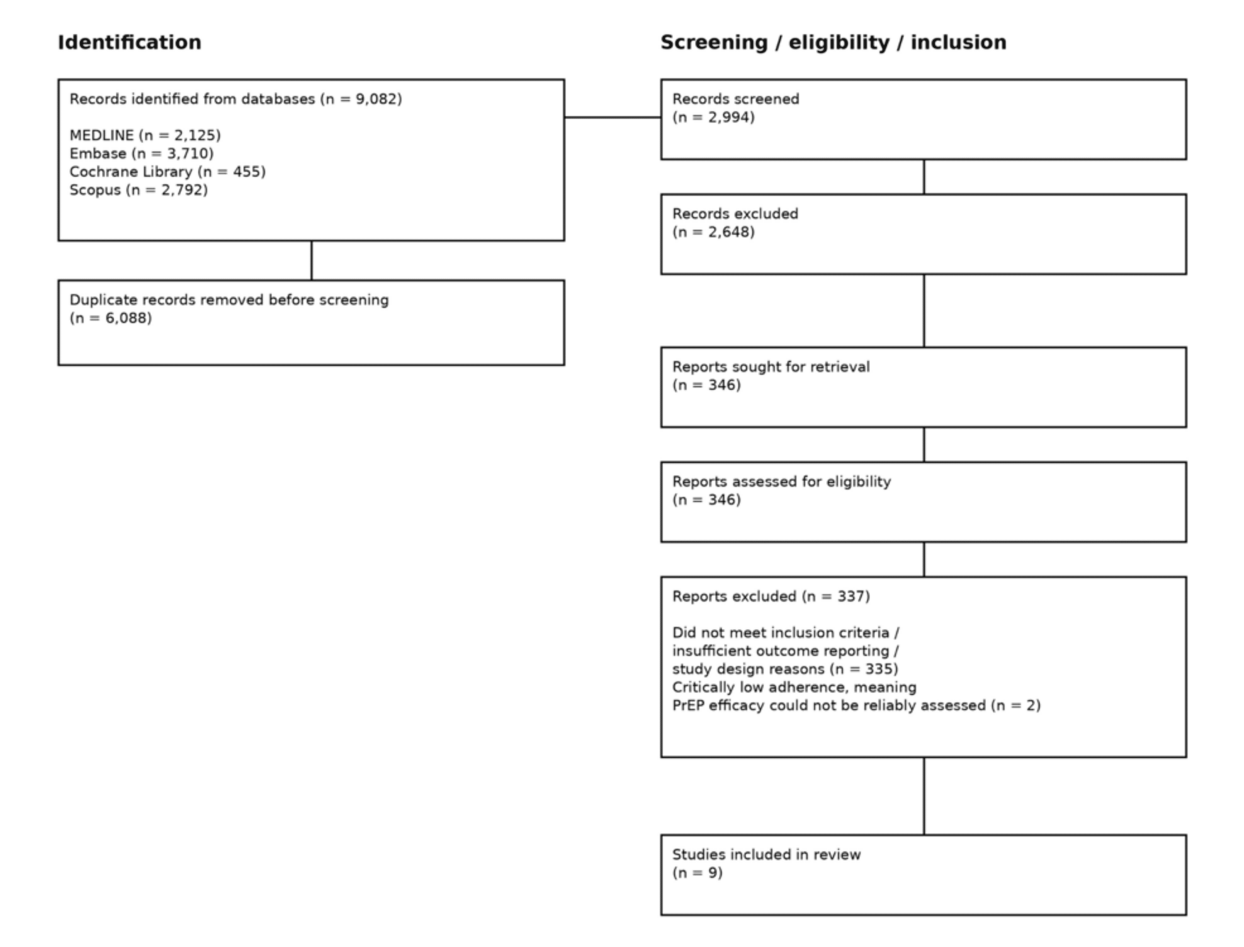
PRISMA flow diagram of study selection Flow diagram illustrating the identification, screening, eligibility assessment, and final inclusion of randomized controlled trials evaluating HIV PrEP. PRISMA: Preferred Reporting Items for Systematic Reviews and Meta-Analyses; HIV: human immunodeficiency virus; PrEP: pre-exposure prophylaxis

The study selection process is illustrated in the PRISMA flow diagram (Figure 1).

Data Analysis

A formal meta-analysis was not conducted due to substantial clinical and methodological heterogeneity across studies, including differences in populations, interventions, adherence measures, and outcome reporting. These factors limit the validity of pooled estimates; therefore, a structured narrative synthesis was undertaken.

To illustrate the relationship between adherence and observed HIV risk reduction across trials, adherence estimates reported in the included studies were plotted against the corresponding intention-to-treat (ITT) risk reduction values. Adherence measures varied across studies and included pharmacokinetic drug levels, pill counts, self-reported adherence, or clinic-based injection attendance for long-acting regimens. Because of heterogeneity in adherence measurement and study populations, the figure is intended as a descriptive visualization rather than a formal meta-regression analysis.

Risk of Bias Assessment

Risk of bias was assessed using the Cochrane Risk of Bias 2 (RoB 2) tool [13] for RCTs, considering randomization, deviations from intended interventions, missing outcome data, outcome measurement, and selective reporting. The final included trials collectively represent over 20,000 participants across multiple continents and diverse high-risk populations, strengthening the generalizability of the synthesis.

Results

Study Selection

A total of 9,082 records were identified across databases; 6,088 duplicates were removed, and 2,994 records were screened by title and abstract. Three hundred and forty-six full-text articles were assessed. Eleven studies initially met the inclusion criteria; two RCTs [14,15] were excluded from the primary efficacy synthesis due to pharmacokinetically confirmed low adherence (24% and 29%, respectively), which limited the interpretability of true biological efficacy. Nine studies were included in the final synthesis.

Risk of Bias Assessment

The overall risk of bias was low across most included RCTs, reflecting robust randomization and outcome ascertainment. One trial by Choopanya et al. [6] raised some concerns related to allocation concealment reporting and potential selection bias given recruitment from PWID in Bangkok; however, it was retained due to relevance to an important high-risk population and because primary outcomes were laboratory-confirmed.

Study Characteristics

Included trials were conducted across Africa, Asia, Europe, North America, and multinational settings and enrolled heterogeneous high-risk populations. PrEP-to-placebo trials included Baeten et al. [7] in Kenya and Uganda among heterosexual serodiscordant couples (TDF arm n=1,219; TDF-FTC arm n=1,217), Choopanya et al. [6] in Thailand among PWID (n=2,413), Grant et al. [5] in a multinational cohort of MSM and transgender participants (n=2,499), McCormack et al. [16] in the United Kingdom among MSM (n=544; immediate vs. deferred PrEP), Molina et al. [17] in France and Canada among MSM (n=400; event-driven PrEP), and Thigpen et al. [18] in Botswana among heterosexual men and women (n=1,219). Comparative regimen trials included Landovitz et al. [8] (HPTN 083) among MSM and transgender women (n=4,566; CAB-LA vs. oral TDF-FTC), Delany-Moretlwe et al. [9] (HPTN 084) among cisgender women in sub-Saharan Africa (n=3,224; CAB-LA vs. oral TDF-FTC), and Ogbuagu et al. [19] (n=5,387; TAF-FTC vs. TDF-FTC). HIV seroconversion was confirmed using laboratory antibody testing with confirmatory assays and/or RNA testing, and incidence was typically reported per 100 person-years (PY). Adherence assessment varied by study, including pharmacokinetic plasma drug levels, pill counts, self-report, and directly observed injection visits for CAB-LA (Table 1).

**Table 1:** Characteristics of the studies included in the systematic review evaluating PrEP effectiveness PrEP: pre-exposure prophylaxis; MSM: men who have sex with men; PWID: people who inject drugs; TDF: tenofovir disoproxil fumarate; FTC: emtricitabine; TAF: tenofovir alafenamide; CAB-LA: long-acting injectable cabotegravir; RCT: randomized controlled trial

Study	Setting	Design	Sample size	Population	PrEP regimen
Grant et al. [5]	Multinational	RCT	2,499	MSM and transgender people	TDF
Choopanya et al. [6]	Thailand	RCT	2,413	PWID	TDF
Baeten et al. [7]	Kenya, Uganda	RCT	1,217	Heterosexual	TDF or TDF-FTC
Landovitz et al. [8]	Multinational	RCT	4,566	MSM and transgender women	CAB-LA
Delany-Moretlwe et al. [9]	Sub-Saharan Africa	RCT	3,224	Cisgender women in sub-Saharan Africa	CAB-LA
McCormack et al. [16]	United Kingdom	RCT	544	MSM	TDF-FTC
Molina et al. [17]	France, Canada	RCT	400	MSM	TDF-FTC
Thigpen et al. [18]	Botswana	RCT	1,219	Heterosexual men and women	TDF-FTC
Ogbuagu et al. [19]	Multinational	RCT	5,387	MSM	TAF-FTC

Outcomes

Efficacy of Oral PrEP in Placebo/Deferred PrEP Trials

Across oral tenofovir-based PrEP trials, HIV incidence reductions were substantial but variable, reflecting differences in populations, background incidence, adherence, and trial design. In the Partners PrEP study [7] among heterosexual serodiscordant couples in Kenya and Uganda, HIV incidence was 0.65 per 100 PY with TDF versus 1.99 per 100 PY with placebo (67% risk reduction). In the same trial, TDF-FTC further reduced incidence to 0.48 per 100 PY compared with 1.99 per 100 PY with placebo (76% risk reduction). Efficacy was strongest in participants with high adherence based on plasma drug concentrations.

In the Bangkok Tenofovir Study [6] among PWID in Thailand, HIV incidence was 0.35 per 100 PY in the PrEP group versus 0.68 per 100 PY in the placebo group (49% risk reduction), with adherence measured via pill counts and plasma levels. In iPrEx [5], the first major TDF-FTC PrEP trial among MSM, incidence was 1.2 per 100 PY with PrEP versus 3.9 per 100 PY with placebo, corresponding to a 44% risk reduction in ITT analysis and a 92% adjusted risk reduction among participants with detectable drug levels. In the PROUD trial [16] conducted in the United Kingdom among MSM using an immediate-versus-deferred design, HIV incidence was 1.2 per 100 PY in the immediate PrEP group and 9.0 per 100 PY in the deferred group, indicating an 86% reduction. In IPERGAY [17], among MSM in France and Canada, event-driven dosing achieved an incidence of 0.91 per 100 PY versus 6.6 per 100 PY with placebo (86% risk reduction), supporting that intermittent dosing can be highly effective in appropriate populations. In Botswana [18], TDF-FTC resulted in 1.2 per 100 PY versus 3.1 per 100 PY with placebo (62% risk reduction), with sex differences in efficacy attributed primarily to adherence.

Comparative Efficacy of CAB-LA and Newer Oral Regimens

Three large comparative trials evaluated newer PrEP modalities or formulations against oral tenofovir-based regimens [8,9,19]. In HPTN 083, CAB-LA demonstrated superior efficacy to daily oral TDF-FTC among cisgender men and transgender women, with HIV incidence of 0.41 per 100 PY in the CAB-LA arm versus 1.22 per 100 PY in the TDF-FTC arm (66% risk reduction) [8]. High observed adherence was consistent with clinic-based injection delivery. In HPTN 084 among cisgender women in sub-Saharan Africa, incidence was 0.20 per 100 PY with CAB-LA versus 1.85 per 100 PY with oral TDF-FTC, representing an 89% risk reduction [9]. In DISCOVER, TAF-FTC was compared with TDF-FTC among MSM and demonstrated non-inferior protection, with a reported incidence of 0.16 per 100 PY (TAF-FTC) versus 0.34 per 100 PY (TDF-FTC), alongside improved renal and bone safety signals with TAF-FTC [19].

Adherence and HIV Risk Reduction

Across trials with pharmacokinetic monitoring, higher adherence consistently corresponded to higher observed efficacy. For example, iPrEx reported a 44% risk reduction in ITT analysis, compared with 92% efficacy among participants with detectable drug levels [5]. Similarly, trials reporting high-adherence subgroup effects demonstrated substantially greater risk reduction, reaching up to approximately 99% in some analyses [7].

**Figure 2:**
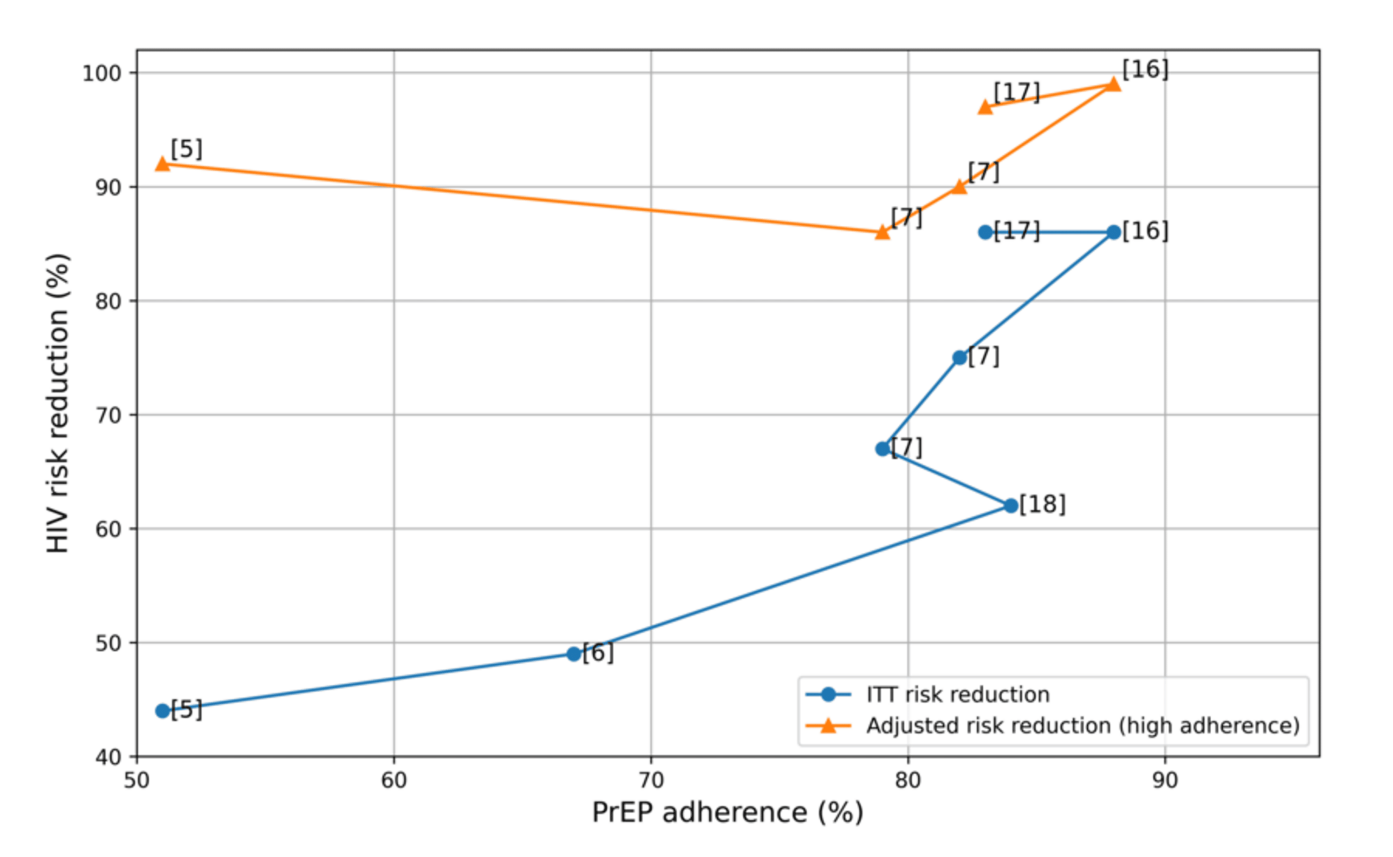
Relationship between adherence and HIV risk reduction Association between adherence (%) and HIV risk reduction across selected randomized controlled trials evaluating oral PrEP. The circular solid line represents the ITT risk reduction. The triangular solid line represents the adjusted risk reduction in high-adherence subgroups. The dashed line shows the linear regression model demonstrating the positive correlation between adherence and ITT risk reduction. HIV: human immunodeficiency virus; PrEP: pre-exposure prophylaxis; ITT: intention-to-treat

Figure 2 presents a descriptive visualization of the relationship between adherence and observed HIV risk reduction across the included trials.

Adverse Events and Risk Compensation

Across oral and injectable PrEP regimens, adverse events were generally mild to moderate and consistent with known drug profiles. In the trial by Baeten et al., nausea, headache, and dizziness occurred in both the TDF and TDF-FTC arms (approximately 23% and 30%, respectively), with higher creatinine values observed in the TDF-FTC arm [7]. Condomless sex decreased from 27% to 13% during follow-up, with no evidence of increased risk behavior. Choopanya et al. reported mild nausea and vomiting and alanine aminotransferase (ALT) elevations, without significant increases in high-risk injecting or sexual behaviors [6]. In iPrEx, nausea (9%), unintentional weight loss, and creatinine elevations were observed, while behavioral measures suggested no increase in risky sexual practices [5]. Thigpen et al. reported nausea, vomiting, dizziness, and ALT/creatinine elevations, with no increase in risky sexual behavior [18].

Molina et al. reported that gastrointestinal symptoms occurred in 7% of TDF users and 2% discontinued due to adverse events; condomless sex decreased slightly, and STI rates remained stable [17]. McCormack et al. reported that nausea/vomiting and renal function abnormalities were noted, condom use remained consistent, and high-risk behavior did not increase [16]. Ogbuagu et al. observed that nausea and renal effects were more frequent with TDF than TAF, with no significant increase in sexual risk behavior across arms [19]. For CAB-LA, injection-site reactions were common; systemic symptoms, including fever, fatigue, and headaches, were reported more frequently in CAB-LA arms in HPTN 083/084, but STI rates and sexual risk behavior remained stable, indicating no consistent evidence of risk compensation [8,9,16].

Discussion

This review synthesizes evidence from nine RCTs evaluating PrEP efficacy across diverse global populations and settings. The findings consistently demonstrate high biological efficacy in preventing HIV transmission; however, the magnitude of protection is strongly influenced by adherence and the specific regimen employed.

Risk reduction ranged from moderate to very high. Grant et al. [5] and McCormack et al. [16] reported particularly high reductions in the context of strong adherence, whereas Choopanya et al. [6], involving PWID in Thailand, demonstrated a lower 49% risk reduction, likely attributable to reduced adherence. Trials evaluating CAB-LA, specifically HPTN 083 and HPTN 084, reported superior efficacy compared with oral regimens [8,9]. Injectable administration mitigates daily pill-taking challenges by maintaining sustained therapeutic drug levels [8,9]. Additionally, the TAF-TDF trial demonstrating non-inferiority reinforces the biological effectiveness of oral PrEP regimens [19].

In relation to the UNAIDS 2030 goal, PrEP's biological potential is clear. However, translating this potential into population-level impact requires optimized adherence and effective delivery systems.

Biological Efficacy Versus Real-World Effectiveness

High-adherence subgroups achieved substantially greater efficacy, confirming PrEP's strong biological effectiveness. In contrast, ITT estimates reflect real-world variability in adherence and persistence.

Grant et al. [5] demonstrated a marked difference between 44% ITT efficacy and 92% efficacy among participants with detectable drug levels, underscoring the necessity of consistent pharmacological exposure. Similar patterns were observed across trials. The FEM-PrEP trial [15] reported adherence of 24% and was discontinued, while the VOICE trial [14] documented adherence of 29%. Both trials highlighted how stigma, geographic context, and relationship dynamics limited adherence and masked PrEP's true biological efficacy [14,15].

The divergence between ideal clinical conditions and real-world implementation explains the efficacy-effectiveness gap. Achieving zero HIV transmissions by 2030, therefore, requires greater attention to social and structural determinants influencing PrEP access and sustained use.

Uptake and Coverage of PrEP

Since 2015, the World Health Organization has recommended PrEP for individuals at substantial risk of HIV [20]. Despite this, uptake and persistence remain below projected targets.

Approximately 590,000 individuals initiated PrEP in 2019 compared with none in 2012; however, global coverage fell short of the three million targets for 2020. By 2023, an estimated 3.5 million individuals were using PrEP, substantially below the UNAIDS 2025 target of 21.2 million [21]. Coverage remains concentrated in parts of North America and selected countries in sub-Saharan Africa. Regions with high PrEP availability have reported significant reductions in HIV incidence among high-risk groups, yet uneven distribution leaves lower-income countries particularly vulnerable. Meeting the UNAIDS 2030 goal will require a large-scale expansion of equitable access.

Structural and Social Determinants

Structural and social factors significantly limit PrEP's real-world effectiveness. Although subgroup analyses demonstrate efficacy exceeding 90% among adherent users, replicating these outcomes at scale is constrained by healthcare access barriers, educational gaps, stigma, and criminalization.

Healthcare system limitations, including provider shortages and prolonged waiting times, impair the initiation and continuity of PrEP [22]. Limited HIV awareness and persistent stigma deter uptake, particularly in sub-Saharan Africa, where fears of being perceived as promiscuous contribute to discontinuation. Criminalization of marginalized groups, including MSM, female sex workers, and PWID, further restricts access to prevention services.

Disparities between developed and developing countries remain evident. Oral PrEP use in low- and middle-income countries (LMICs) represented only 28% of the UNAIDS target of three million people. Moreover, earlier trials underrepresented several high-risk populations, including transgender women and sex workers, who experience substantial structural barriers.

The Potential of Long-Acting Injectable PrEP

CAB-LA offers a strategy to overcome adherence challenges associated with daily oral regimens. HPTN 083 demonstrated that CAB-LA administered every eight weeks reduced HIV incidence by 66% relative to daily oral TDF-FTC among MSM and transgender women. HPTN 084 reported nearly 90% lower HIV incidence among African women receiving CAB-LA compared with oral PrEP. By maintaining sustained drug levels, injectable regimens reduce reliance on daily pill-taking and improve adherence [8,9].

CAB-LA has been reported as a preferred modality in certain high-risk populations due to its convenience and discreet nature [9], addressing both adherence and stigma-related challenges. However, implementation barriers remain substantial. CAB-LA has been priced at approximately $3,700 per dose in the United States, equating to around $22,000 per person annually, far exceeding the cost of standard oral regimens. Additionally, fear of needles and injection-site reactions may limit acceptability. While CAB-LA has transformative potential, financial and logistical constraints currently restrict accessibility, particularly in developing countries. Cost reduction and expanded access will be essential if CAB-LA is to contribute meaningfully to achieving the UNAIDS 2030 targets.

Limitations

This review has a few limitations. First, the evidence base was dominated by RCTs and comparative trials; real-world implementation data were not synthesized in depth, potentially limiting inference about routine program performance. Second, heterogeneity in populations, adherence assessment, and intervention modalities precluded meta-analysis and pooled effect estimation. Third, restricting inclusion to English-language publications may have excluded relevant studies.

Some key populations also remain underrepresented in the available trial evidence, including female sex workers, certain transgender communities, and settings with concentrated epidemics outside Africa, Europe, and North America. Publication bias cannot be excluded, as studies demonstrating significant protective effects of PrEP may be more likely to be published than those reporting null findings.

Finally, screening and eligibility assessment were primarily conducted by a single reviewer, which may introduce potential selection bias; however, key inclusion decisions were reviewed with a senior academic supervisor.

## Conclusions

PrEP is a highly effective biomedical strategy for preventing HIV acquisition across diverse populations when implemented appropriately. Evidence from RCTs consistently demonstrates its capacity to substantially reduce HIV transmission under conditions of adequate adherence and program support. However, the effectiveness of PrEP in real-world settings depends on sustained engagement in prevention services, equitable access, and the ability of health systems to support consistent use.

Maximizing the public health impact of PrEP will require continued efforts to strengthen implementation strategies, improve accessibility, and support long-term adherence. Expanding prevention delivery models and integrating newer prevention modalities may further enhance the effectiveness of HIV prevention programs.
